# Supplementary figures and images for: Species clustering, climate effects, and introduced species in 5 million city trees across 63 US cities (part 2 of 2)
Source: eLife. 2022 Sep 27;11:e77891. doi: 10.7554/eLife.77891 (PMC9578703; doi:10.7554/eLife.77891)

# Rochester

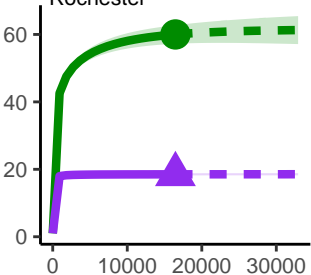

Supplement: Figure 2—source data 1. — This zipped file includes plots for the tree community of each city, showing rarefaction curves as calculated by the R package iNext. Each city includes a plot for all trees and a plot for all naturally occurring trees. [file elife-77891-fig2-data1.zip › Rarefaction_Plots/Rochester_RarefactionPlot_Diversity_native.pdf]

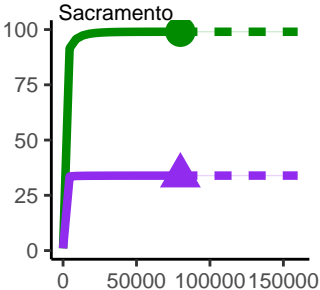

Supplement: Figure 2—source data 1. — This zipped file includes plots for the tree community of each city, showing rarefaction curves as calculated by the R package iNext. Each city includes a plot for all trees and a plot for all naturally occurring trees. [file elife-77891-fig2-data1.zip › Rarefaction_Plots/Sacramento_RarefactionPlot_Diversity_all.pdf]

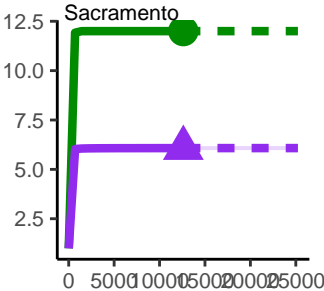

Supplement: Figure 2—source data 1. — This zipped file includes plots for the tree community of each city, showing rarefaction curves as calculated by the R package iNext. Each city includes a plot for all trees and a plot for all naturally occurring trees. [file elife-77891-fig2-data1.zip › Rarefaction_Plots/Sacramento_RarefactionPlot_Diversity_native.pdf]

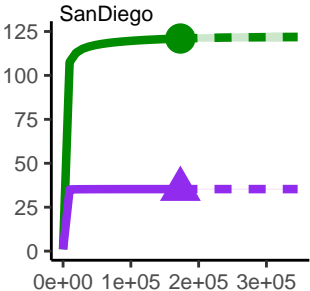

Supplement: Figure 2—source data 1. — This zipped file includes plots for the tree community of each city, showing rarefaction curves as calculated by the R package iNext. Each city includes a plot for all trees and a plot for all naturally occurring trees. [file elife-77891-fig2-data1.zip › Rarefaction_Plots/San Diego_RarefactionPlot_Diversity_all.pdf]

SanDiego

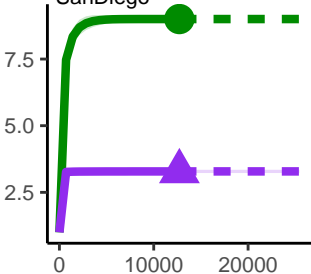

Supplement: Figure 2—source data 1. — This zipped file includes plots for the tree community of each city, showing rarefaction curves as calculated by the R package iNext. Each city includes a plot for all trees and a plot for all naturally occurring trees. [file elife-77891-fig2-data1.zip › Rarefaction_Plots/San Diego_RarefactionPlot_Diversity_native.pdf]

# San Francisco

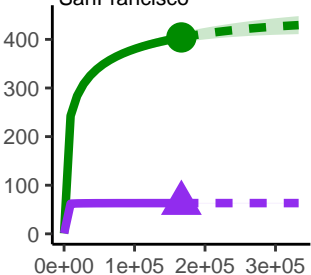

Supplement: Figure 2—source data 1. — This zipped file includes plots for the tree community of each city, showing rarefaction curves as calculated by the R package iNext. Each city includes a plot for all trees and a plot for all naturally occurring trees. [file elife-77891-fig2-data1.zip › Rarefaction_Plots/San Francisco_RarefactionPlot_Diversity_all.pdf]

# San Francisco

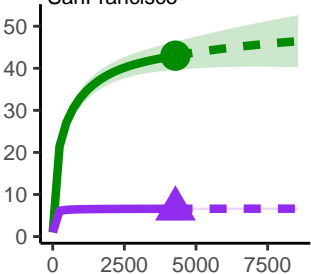

Supplement: Figure 2—source data 1. — This zipped file includes plots for the tree community of each city, showing rarefaction curves as calculated by the R package iNext. Each city includes a plot for all trees and a plot for all naturally occurring trees. [file elife-77891-fig2-data1.zip › Rarefaction_Plots/San Francisco_RarefactionPlot_Diversity_native.pdf]

SanJose

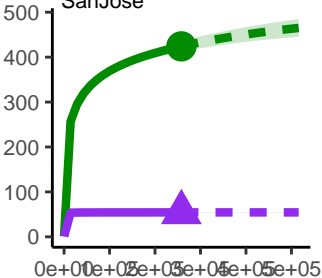

Supplement: Figure 2—source data 1. — This zipped file includes plots for the tree community of each city, showing rarefaction curves as calculated by the R package iNext. Each city includes a plot for all trees and a plot for all naturally occurring trees. [file elife-77891-fig2-data1.zip › Rarefaction_Plots/San Jose_RarefactionPlot_Diversity_all.pdf]

# SanJose

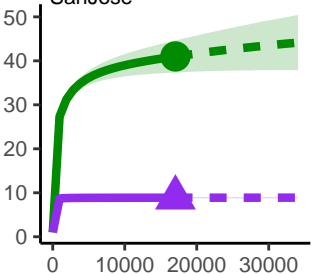

Supplement: Figure 2—source data 1. — This zipped file includes plots for the tree community of each city, showing rarefaction curves as calculated by the R package iNext. Each city includes a plot for all trees and a plot for all naturally occurring trees. [file elife-77891-fig2-data1.zip › Rarefaction_Plots/San Jose_RarefactionPlot_Diversity_native.pdf]

# SantaRosa

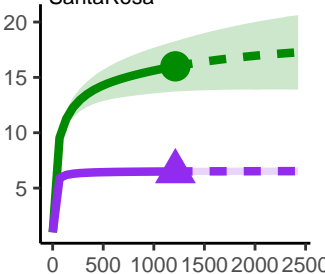

Supplement: Figure 2—source data 1. — This zipped file includes plots for the tree community of each city, showing rarefaction curves as calculated by the R package iNext. Each city includes a plot for all trees and a plot for all naturally occurring trees. [file elife-77891-fig2-data1.zip › Rarefaction_Plots/Santa Rosa_RarefactionPlot_Diversity_all.pdf]

Seattle

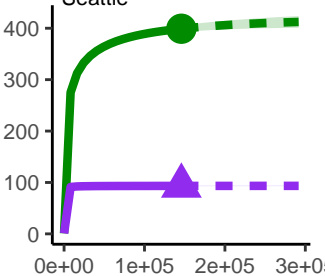

Supplement: Figure 2—source data 1. — This zipped file includes plots for the tree community of each city, showing rarefaction curves as calculated by the R package iNext. Each city includes a plot for all trees and a plot for all naturally occurring trees. [file elife-77891-fig2-data1.zip › Rarefaction_Plots/Seattle_RarefactionPlot_Diversity_all.pdf]

Seattle

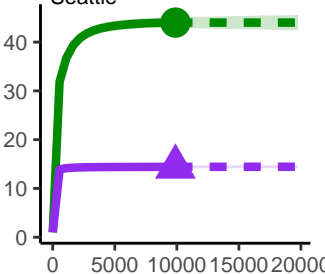

Supplement: Figure 2—source data 1. — This zipped file includes plots for the tree community of each city, showing rarefaction curves as calculated by the R package iNext. Each city includes a plot for all trees and a plot for all naturally occurring trees. [file elife-77891-fig2-data1.zip › Rarefaction_Plots/Seattle_RarefactionPlot_Diversity_native.pdf]

# Sioux Falls

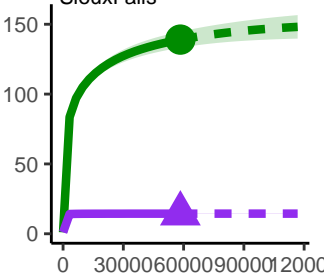

Supplement: Figure 2—source data 1. — This zipped file includes plots for the tree community of each city, showing rarefaction curves as calculated by the R package iNext. Each city includes a plot for all trees and a plot for all naturally occurring trees. [file elife-77891-fig2-data1.zip › Rarefaction_Plots/Sioux Falls_RarefactionPlot_Diversity_all.pdf]

# Sioux Falls

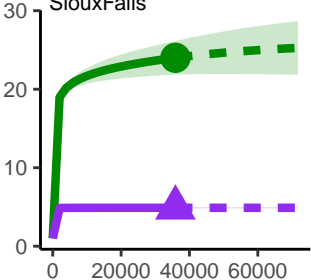

Supplement: Figure 2—source data 1. — This zipped file includes plots for the tree community of each city, showing rarefaction curves as calculated by the R package iNext. Each city includes a plot for all trees and a plot for all naturally occurring trees. [file elife-77891-fig2-data1.zip › Rarefaction_Plots/Sioux Falls_RarefactionPlot_Diversity_native.pdf]

StLouis

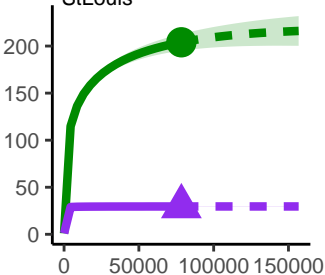

Supplement: Figure 2—source data 1. — This zipped file includes plots for the tree community of each city, showing rarefaction curves as calculated by the R package iNext. Each city includes a plot for all trees and a plot for all naturally occurring trees. [file elife-77891-fig2-data1.zip › Rarefaction_Plots/St. Louis_RarefactionPlot_Diversity_all.pdf]

StLouis

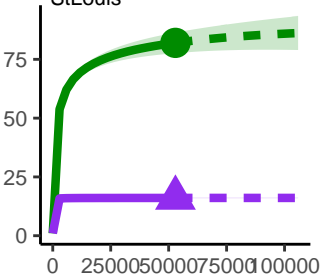

Supplement: Figure 2—source data 1. — This zipped file includes plots for the tree community of each city, showing rarefaction curves as calculated by the R package iNext. Each city includes a plot for all trees and a plot for all naturally occurring trees. [file elife-77891-fig2-data1.zip › Rarefaction_Plots/St. Louis_RarefactionPlot_Diversity_native.pdf]

Stockton

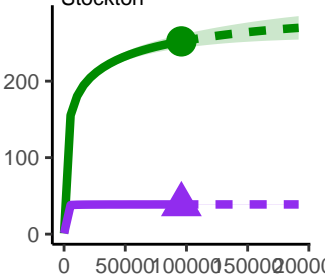

Supplement: Figure 2—source data 1. — This zipped file includes plots for the tree community of each city, showing rarefaction curves as calculated by the R package iNext. Each city includes a plot for all trees and a plot for all naturally occurring trees. [file elife-77891-fig2-data1.zip › Rarefaction_Plots/Stockton_RarefactionPlot_Diversity_all.pdf]

# Stockton

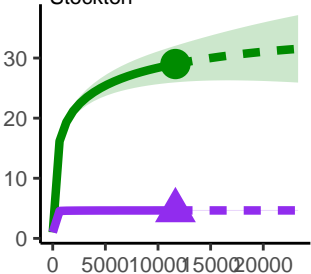

Supplement: Figure 2—source data 1. — This zipped file includes plots for the tree community of each city, showing rarefaction curves as calculated by the R package iNext. Each city includes a plot for all trees and a plot for all naturally occurring trees. [file elife-77891-fig2-data1.zip › Rarefaction_Plots/Stockton_RarefactionPlot_Diversity_native.pdf]

Tampa

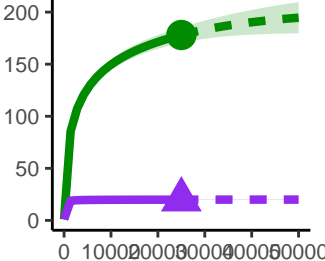

Supplement: Figure 2—source data 1. — This zipped file includes plots for the tree community of each city, showing rarefaction curves as calculated by the R package iNext. Each city includes a plot for all trees and a plot for all naturally occurring trees. [file elife-77891-fig2-data1.zip › Rarefaction_Plots/Tampa_RarefactionPlot_Diversity_all.pdf]

Tampa

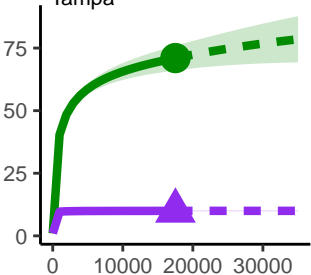

Supplement: Figure 2—source data 1. — This zipped file includes plots for the tree community of each city, showing rarefaction curves as calculated by the R package iNext. Each city includes a plot for all trees and a plot for all naturally occurring trees. [file elife-77891-fig2-data1.zip › Rarefaction_Plots/Tampa_RarefactionPlot_Diversity_native.pdf]

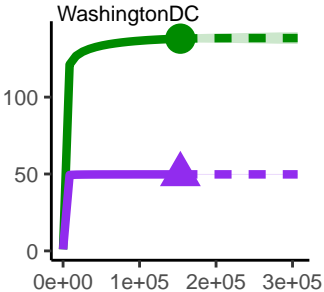

Supplement: Figure 2—source data 1. — This zipped file includes plots for the tree community of each city, showing rarefaction curves as calculated by the R package iNext. Each city includes a plot for all trees and a plot for all naturally occurring trees. [file elife-77891-fig2-data1.zip › Rarefaction_Plots/Washington (DC)_RarefactionPlot_Diversity_all.pdf]

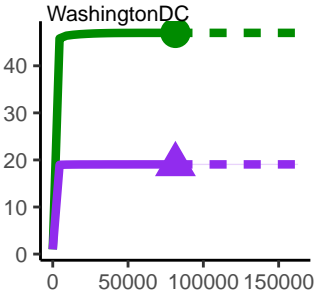

Supplement: Figure 2—source data 1. — This zipped file includes plots for the tree community of each city, showing rarefaction curves as calculated by the R package iNext. Each city includes a plot for all trees and a plot for all naturally occurring trees. [file elife-77891-fig2-data1.zip › Rarefaction_Plots/Washington (DC)_RarefactionPlot_Diversity_native.pdf]

## Worcester

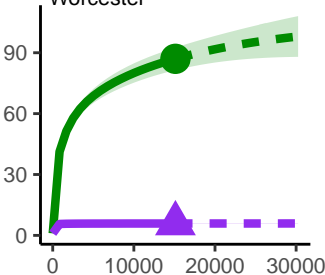

Supplement: Figure 2—source data 1. — This zipped file includes plots for the tree community of each city, showing rarefaction curves as calculated by the R package iNext. Each city includes a plot for all trees and a plot for all naturally occurring trees. [file elife-77891-fig2-data1.zip › Rarefaction_Plots/Worcester_RarefactionPlot_Diversity_all.pdf]

# Worcester

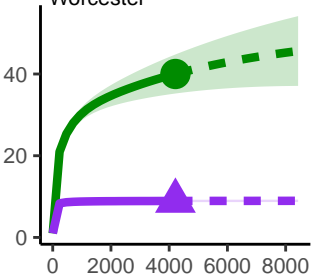

Supplement: Figure 2—source data 1. — This zipped file includes plots for the tree community of each city, showing rarefaction curves as calculated by the R package iNext. Each city includes a plot for all trees and a plot for all naturally occurring trees. [file elife-77891-fig2-data1.zip › Rarefaction_Plots/Worcester_RarefactionPlot_Diversity_native.pdf]
