# Supplementary figures and images for: Species clustering, climate effects, and introduced species in 5 million city trees across 63 US cities (part 1 of 2)
Source: eLife. 2022 Sep 27;11:e77891. doi: 10.7554/eLife.77891 (PMC9578703; doi:10.7554/eLife.77891)

# Albuquerque

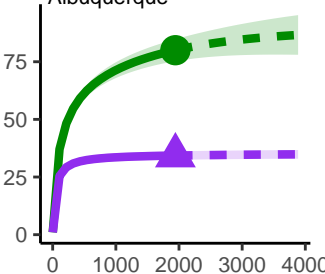

Supplement: Figure 2—source data 1. — This zipped file includes plots for the tree community of each city, showing rarefaction curves as calculated by the R package iNext. Each city includes a plot for all trees and a plot for all naturally occurring trees. [file elife-77891-fig2-data1.zip › Rarefaction_Plots/Albuquerque_RarefactionPlot_Diversity_all.pdf]

# Albuquerque

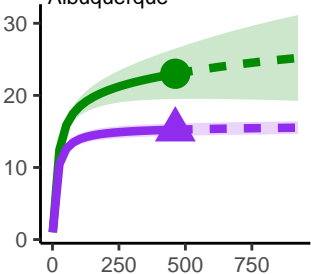

Supplement: Figure 2—source data 1. — This zipped file includes plots for the tree community of each city, showing rarefaction curves as calculated by the R package iNext. Each city includes a plot for all trees and a plot for all naturally occurring trees. [file elife-77891-fig2-data1.zip › Rarefaction_Plots/Albuquerque_RarefactionPlot_Diversity_native.pdf]

Anaheim

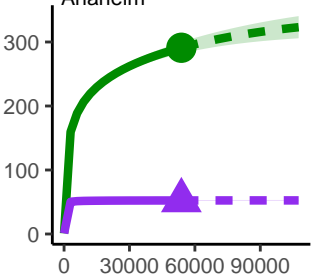

Supplement: Figure 2—source data 1. — This zipped file includes plots for the tree community of each city, showing rarefaction curves as calculated by the R package iNext. Each city includes a plot for all trees and a plot for all naturally occurring trees. [file elife-77891-fig2-data1.zip › Rarefaction_Plots/Anaheim_RarefactionPlot_Diversity_all.pdf]

# Anaheim

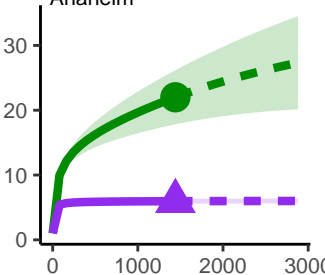

Supplement: Figure 2—source data 1. — This zipped file includes plots for the tree community of each city, showing rarefaction curves as calculated by the R package iNext. Each city includes a plot for all trees and a plot for all naturally occurring trees. [file elife-77891-fig2-data1.zip › Rarefaction_Plots/Anaheim_RarefactionPlot_Diversity_native.pdf]

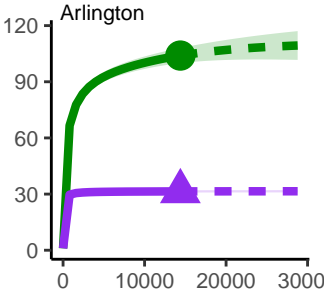

Supplement: Figure 2—source data 1. — This zipped file includes plots for the tree community of each city, showing rarefaction curves as calculated by the R package iNext. Each city includes a plot for all trees and a plot for all naturally occurring trees. [file elife-77891-fig2-data1.zip › Rarefaction_Plots/Arlington_RarefactionPlot_Diversity_all.pdf]

Arlington

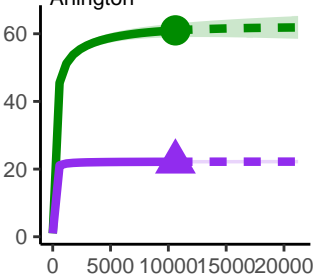

Supplement: Figure 2—source data 1. — This zipped file includes plots for the tree community of each city, showing rarefaction curves as calculated by the R package iNext. Each city includes a plot for all trees and a plot for all naturally occurring trees. [file elife-77891-fig2-data1.zip › Rarefaction_Plots/Arlington_RarefactionPlot_Diversity_native.pdf]

Atlanta

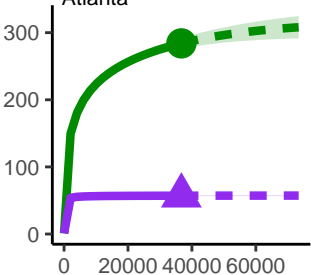

Supplement: Figure 2—source data 1. — This zipped file includes plots for the tree community of each city, showing rarefaction curves as calculated by the R package iNext. Each city includes a plot for all trees and a plot for all naturally occurring trees. [file elife-77891-fig2-data1.zip › Rarefaction_Plots/Atlanta_RarefactionPlot_Diversity_all.pdf]

Atlanta

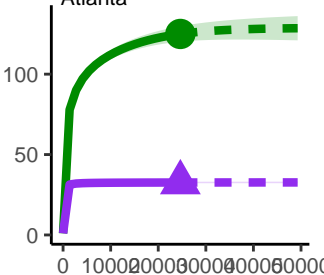

Supplement: Figure 2—source data 1. — This zipped file includes plots for the tree community of each city, showing rarefaction curves as calculated by the R package iNext. Each city includes a plot for all trees and a plot for all naturally occurring trees. [file elife-77891-fig2-data1.zip › Rarefaction_Plots/Atlanta_RarefactionPlot_Diversity_native.pdf]

AuroraCO

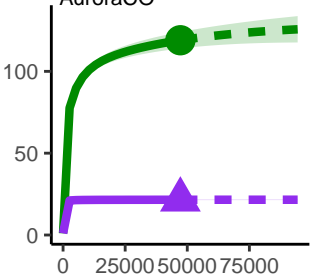

Supplement: Figure 2—source data 1. — This zipped file includes plots for the tree community of each city, showing rarefaction curves as calculated by the R package iNext. Each city includes a plot for all trees and a plot for all naturally occurring trees. [file elife-77891-fig2-data1.zip › Rarefaction_Plots/Aurora (CO)_RarefactionPlot_Diversity_all.pdf]

AuroraCO

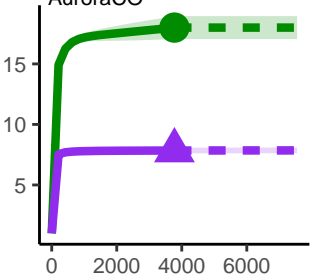

Supplement: Figure 2—source data 1. — This zipped file includes plots for the tree community of each city, showing rarefaction curves as calculated by the R package iNext. Each city includes a plot for all trees and a plot for all naturally occurring trees. [file elife-77891-fig2-data1.zip › Rarefaction_Plots/Aurora (CO)_RarefactionPlot_Diversity_native.pdf]

Austin

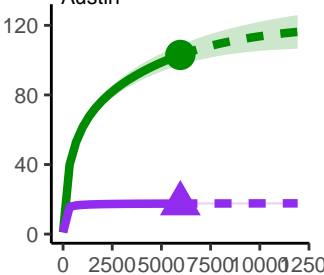

Supplement: Figure 2—source data 1. — This zipped file includes plots for the tree community of each city, showing rarefaction curves as calculated by the R package iNext. Each city includes a plot for all trees and a plot for all naturally occurring trees. [file elife-77891-fig2-data1.zip › Rarefaction_Plots/Austin_RarefactionPlot_Diversity_all.pdf]

Austin

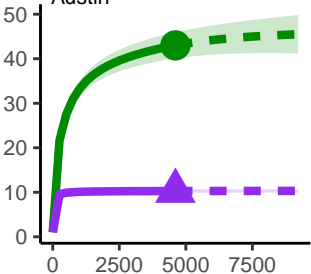

Supplement: Figure 2—source data 1. — This zipped file includes plots for the tree community of each city, showing rarefaction curves as calculated by the R package iNext. Each city includes a plot for all trees and a plot for all naturally occurring trees. [file elife-77891-fig2-data1.zip › Rarefaction_Plots/Austin_RarefactionPlot_Diversity_native.pdf]

# Baltimore

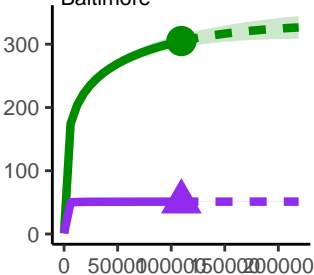

Supplement: Figure 2—source data 1. — This zipped file includes plots for the tree community of each city, showing rarefaction curves as calculated by the R package iNext. Each city includes a plot for all trees and a plot for all naturally occurring trees. [file elife-77891-fig2-data1.zip › Rarefaction_Plots/Baltimore_RarefactionPlot_Diversity_all.pdf]

## Baltimore

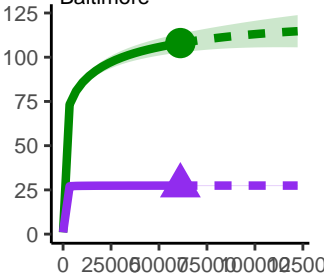

Supplement: Figure 2—source data 1. — This zipped file includes plots for the tree community of each city, showing rarefaction curves as calculated by the R package iNext. Each city includes a plot for all trees and a plot for all naturally occurring trees. [file elife-77891-fig2-data1.zip › Rarefaction_Plots/Baltimore_RarefactionPlot_Diversity_native.pdf]

Boston

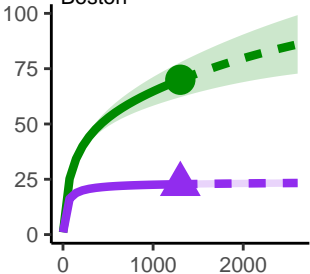

Supplement: Figure 2—source data 1. — This zipped file includes plots for the tree community of each city, showing rarefaction curves as calculated by the R package iNext. Each city includes a plot for all trees and a plot for all naturally occurring trees. [file elife-77891-fig2-data1.zip › Rarefaction_Plots/Boston_RarefactionPlot_Diversity_all.pdf]

## Boston

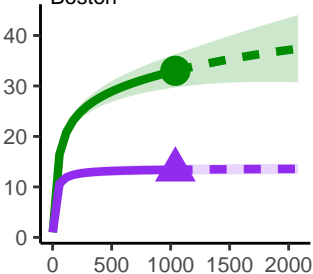

Supplement: Figure 2—source data 1. — This zipped file includes plots for the tree community of each city, showing rarefaction curves as calculated by the R package iNext. Each city includes a plot for all trees and a plot for all naturally occurring trees. [file elife-77891-fig2-data1.zip › Rarefaction_Plots/Boston_RarefactionPlot_Diversity_native.pdf]

# Buffalo

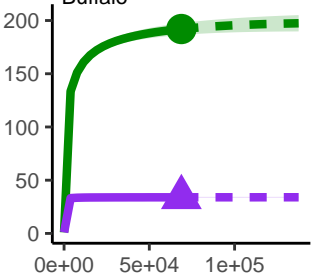

Supplement: Figure 2—source data 1. — This zipped file includes plots for the tree community of each city, showing rarefaction curves as calculated by the R package iNext. Each city includes a plot for all trees and a plot for all naturally occurring trees. [file elife-77891-fig2-data1.zip › Rarefaction_Plots/Buffalo_RarefactionPlot_Diversity_all.pdf]

## Buffalo

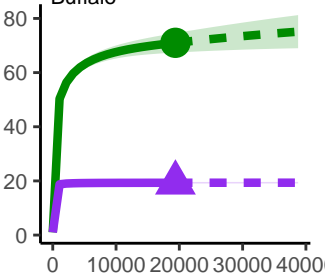

Supplement: Figure 2—source data 1. — This zipped file includes plots for the tree community of each city, showing rarefaction curves as calculated by the R package iNext. Each city includes a plot for all trees and a plot for all naturally occurring trees. [file elife-77891-fig2-data1.zip › Rarefaction_Plots/Buffalo_RarefactionPlot_Diversity_native.pdf]

## CapeCoral

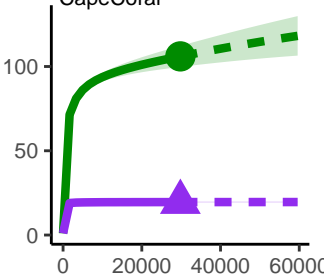

Supplement: Figure 2—source data 1. — This zipped file includes plots for the tree community of each city, showing rarefaction curves as calculated by the R package iNext. Each city includes a plot for all trees and a plot for all naturally occurring trees. [file elife-77891-fig2-data1.zip › Rarefaction_Plots/Cape Coral_RarefactionPlot_Diversity_all.pdf]

CapeCoral

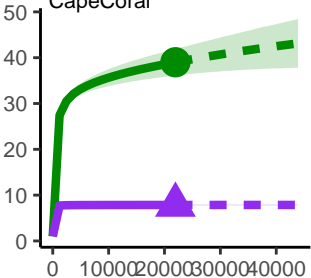

Supplement: Figure 2—source data 1. — This zipped file includes plots for the tree community of each city, showing rarefaction curves as calculated by the R package iNext. Each city includes a plot for all trees and a plot for all naturally occurring trees. [file elife-77891-fig2-data1.zip › Rarefaction_Plots/Cape Coral_RarefactionPlot_Diversity_native.pdf]

# ColoradoSprings

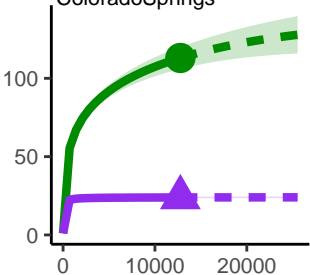

Supplement: Figure 2—source data 1. — This zipped file includes plots for the tree community of each city, showing rarefaction curves as calculated by the R package iNext. Each city includes a plot for all trees and a plot for all naturally occurring trees. [file elife-77891-fig2-data1.zip › Rarefaction_Plots/Colorado Springs_RarefactionPlot_Diversity_all.pdf]

# ColoradoSprings

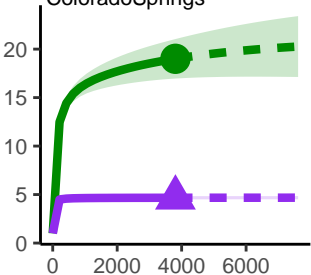

Supplement: Figure 2—source data 1. — This zipped file includes plots for the tree community of each city, showing rarefaction curves as calculated by the R package iNext. Each city includes a plot for all trees and a plot for all naturally occurring trees. [file elife-77891-fig2-data1.zip › Rarefaction_Plots/Colorado Springs_RarefactionPlot_Diversity_native.pdf]

## Columbus

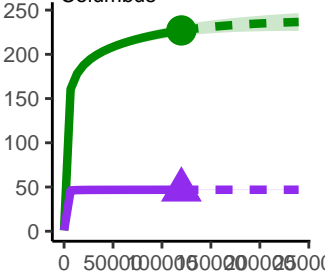

Supplement: Figure 2—source data 1. — This zipped file includes plots for the tree community of each city, showing rarefaction curves as calculated by the R package iNext. Each city includes a plot for all trees and a plot for all naturally occurring trees. [file elife-77891-fig2-data1.zip › Rarefaction_Plots/Columbus_RarefactionPlot_Diversity_all.pdf]

# Columbus

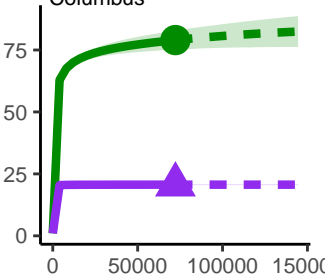

Supplement: Figure 2—source data 1. — This zipped file includes plots for the tree community of each city, showing rarefaction curves as calculated by the R package iNext. Each city includes a plot for all trees and a plot for all naturally occurring trees. [file elife-77891-fig2-data1.zip › Rarefaction_Plots/Columbus_RarefactionPlot_Diversity_native.pdf]

Dallas

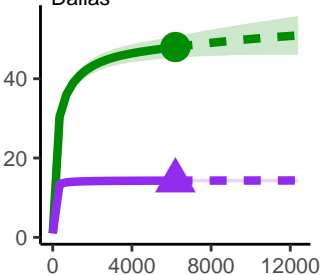

Supplement: Figure 2—source data 1. — This zipped file includes plots for the tree community of each city, showing rarefaction curves as calculated by the R package iNext. Each city includes a plot for all trees and a plot for all naturally occurring trees. [file elife-77891-fig2-data1.zip › Rarefaction_Plots/Dallas_RarefactionPlot_Diversity_all.pdf]

Dallas

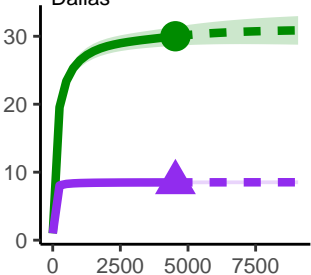

Supplement: Figure 2—source data 1. — This zipped file includes plots for the tree community of each city, showing rarefaction curves as calculated by the R package iNext. Each city includes a plot for all trees and a plot for all naturally occurring trees. [file elife-77891-fig2-data1.zip › Rarefaction_Plots/Dallas_RarefactionPlot_Diversity_native.pdf]

Denver

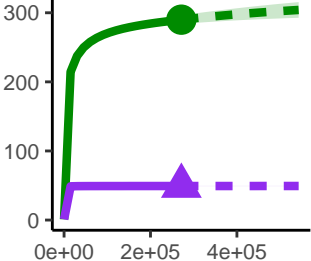

Supplement: Figure 2—source data 1. — This zipped file includes plots for the tree community of each city, showing rarefaction curves as calculated by the R package iNext. Each city includes a plot for all trees and a plot for all naturally occurring trees. [file elife-77891-fig2-data1.zip › Rarefaction_Plots/Denver_RarefactionPlot_Diversity_all.pdf]

Denver

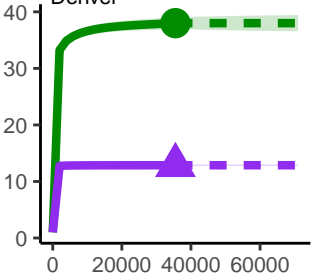

Supplement: Figure 2—source data 1. — This zipped file includes plots for the tree community of each city, showing rarefaction curves as calculated by the R package iNext. Each city includes a plot for all trees and a plot for all naturally occurring trees. [file elife-77891-fig2-data1.zip › Rarefaction_Plots/Denver_RarefactionPlot_Diversity_native.pdf]

Des Moines

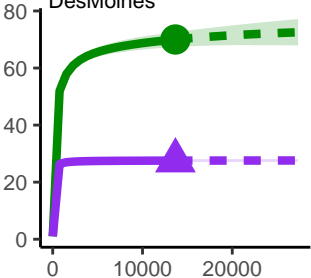

Supplement: Figure 2—source data 1. — This zipped file includes plots for the tree community of each city, showing rarefaction curves as calculated by the R package iNext. Each city includes a plot for all trees and a plot for all naturally occurring trees. [file elife-77891-fig2-data1.zip › Rarefaction_Plots/Des Moines_RarefactionPlot_Diversity_all.pdf]

DesMoines

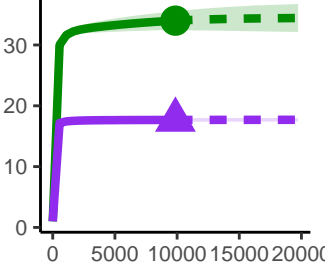

Supplement: Figure 2—source data 1. — This zipped file includes plots for the tree community of each city, showing rarefaction curves as calculated by the R package iNext. Each city includes a plot for all trees and a plot for all naturally occurring trees. [file elife-77891-fig2-data1.zip › Rarefaction_Plots/Des Moines_RarefactionPlot_Diversity_native.pdf]

# Detroit

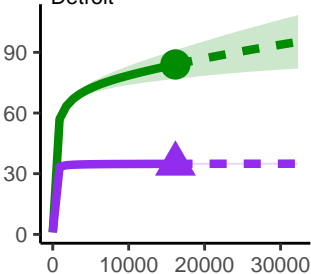

Supplement: Figure 2—source data 1. — This zipped file includes plots for the tree community of each city, showing rarefaction curves as calculated by the R package iNext. Each city includes a plot for all trees and a plot for all naturally occurring trees. [file elife-77891-fig2-data1.zip › Rarefaction_Plots/Detroit_RarefactionPlot_Diversity_all.pdf]

# Detroit

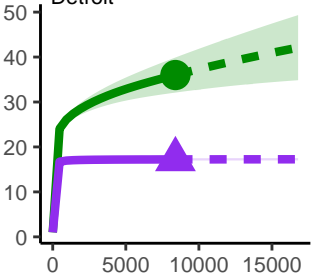

Supplement: Figure 2—source data 1. — This zipped file includes plots for the tree community of each city, showing rarefaction curves as calculated by the R package iNext. Each city includes a plot for all trees and a plot for all naturally occurring trees. [file elife-77891-fig2-data1.zip › Rarefaction_Plots/Detroit_RarefactionPlot_Diversity_native.pdf]

Durham

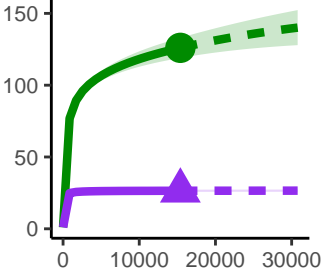

Supplement: Figure 2—source data 1. — This zipped file includes plots for the tree community of each city, showing rarefaction curves as calculated by the R package iNext. Each city includes a plot for all trees and a plot for all naturally occurring trees. [file elife-77891-fig2-data1.zip › Rarefaction_Plots/Durham_RarefactionPlot_Diversity_all.pdf]

Durham

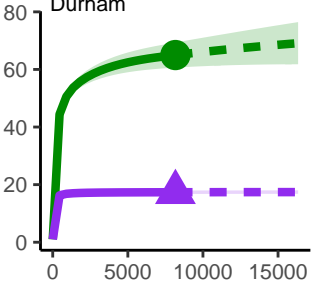

Supplement: Figure 2—source data 1. — This zipped file includes plots for the tree community of each city, showing rarefaction curves as calculated by the R package iNext. Each city includes a plot for all trees and a plot for all naturally occurring trees. [file elife-77891-fig2-data1.zip › Rarefaction_Plots/Durham_RarefactionPlot_Diversity_native.pdf]

Fresno

80  
60  
40  
20  
0

0 2500 5000 7500 10000 12500

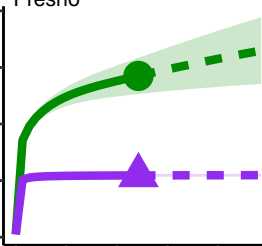

Supplement: Figure 2—source data 1. — This zipped file includes plots for the tree community of each city, showing rarefaction curves as calculated by the R package iNext. Each city includes a plot for all trees and a plot for all naturally occurring trees. [file elife-77891-fig2-data1.zip › Rarefaction_Plots/Fresno_RarefactionPlot_Diversity_all.pdf]

Fresno

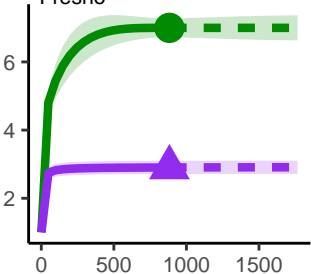

Supplement: Figure 2—source data 1. — This zipped file includes plots for the tree community of each city, showing rarefaction curves as calculated by the R package iNext. Each city includes a plot for all trees and a plot for all naturally occurring trees. [file elife-77891-fig2-data1.zip › Rarefaction_Plots/Fresno_RarefactionPlot_Diversity_native.pdf]

# GardenGrove

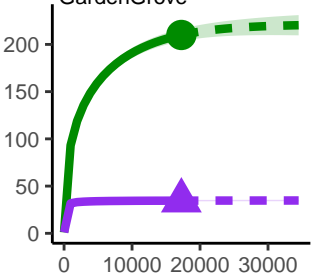

Supplement: Figure 2—source data 1. — This zipped file includes plots for the tree community of each city, showing rarefaction curves as calculated by the R package iNext. Each city includes a plot for all trees and a plot for all naturally occurring trees. [file elife-77891-fig2-data1.zip › Rarefaction_Plots/Garden Grove_RarefactionPlot_Diversity_all.pdf]

# GardenGrove

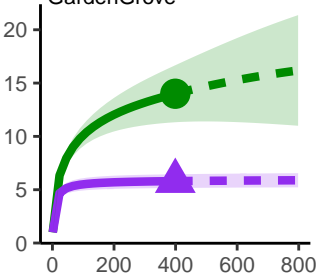

Supplement: Figure 2—source data 1. — This zipped file includes plots for the tree community of each city, showing rarefaction curves as calculated by the R package iNext. Each city includes a plot for all trees and a plot for all naturally occurring trees. [file elife-77891-fig2-data1.zip › Rarefaction_Plots/Garden Grove_RarefactionPlot_Diversity_native.pdf]

# GrandRapids

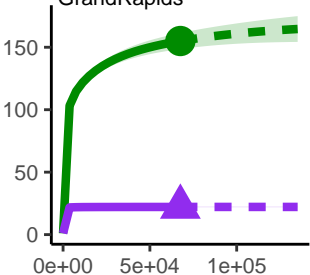

Supplement: Figure 2—source data 1. — This zipped file includes plots for the tree community of each city, showing rarefaction curves as calculated by the R package iNext. Each city includes a plot for all trees and a plot for all naturally occurring trees. [file elife-77891-fig2-data1.zip › Rarefaction_Plots/Grand Rapids_RarefactionPlot_Diversity_all.pdf]

# GrandRapids

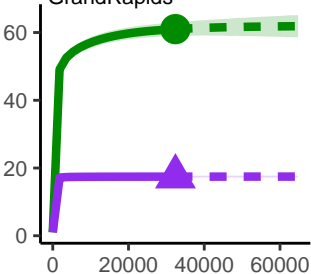

Supplement: Figure 2—source data 1. — This zipped file includes plots for the tree community of each city, showing rarefaction curves as calculated by the R package iNext. Each city includes a plot for all trees and a plot for all naturally occurring trees. [file elife-77891-fig2-data1.zip › Rarefaction_Plots/Grand Rapids_RarefactionPlot_Diversity_native.pdf]

# Greensboro

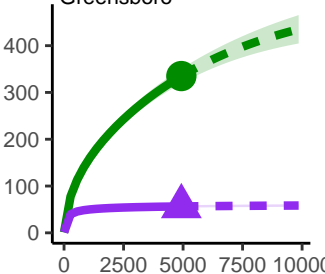

Supplement: Figure 2—source data 1. — This zipped file includes plots for the tree community of each city, showing rarefaction curves as calculated by the R package iNext. Each city includes a plot for all trees and a plot for all naturally occurring trees. [file elife-77891-fig2-data1.zip › Rarefaction_Plots/Greensboro_RarefactionPlot_Diversity_all.pdf]

# Greensboro

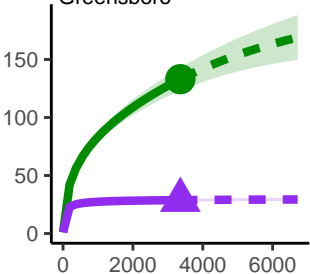

Supplement: Figure 2—source data 1. — This zipped file includes plots for the tree community of each city, showing rarefaction curves as calculated by the R package iNext. Each city includes a plot for all trees and a plot for all naturally occurring trees. [file elife-77891-fig2-data1.zip › Rarefaction_Plots/Greensboro_RarefactionPlot_Diversity_native.pdf]

# Honolulu

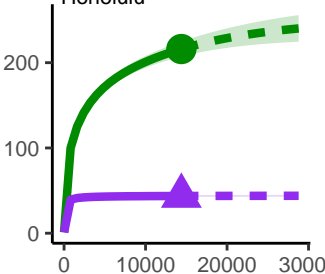

Supplement: Figure 2—source data 1. — This zipped file includes plots for the tree community of each city, showing rarefaction curves as calculated by the R package iNext. Each city includes a plot for all trees and a plot for all naturally occurring trees. [file elife-77891-fig2-data1.zip › Rarefaction_Plots/Honolulu_RarefactionPlot_Diversity_all.pdf]

Honolulu

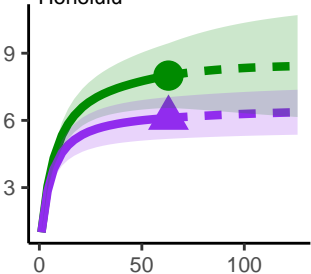

Supplement: Figure 2—source data 1. — This zipped file includes plots for the tree community of each city, showing rarefaction curves as calculated by the R package iNext. Each city includes a plot for all trees and a plot for all naturally occurring trees. [file elife-77891-fig2-data1.zip › Rarefaction_Plots/Honolulu_RarefactionPlot_Diversity_native.pdf]

# Houston

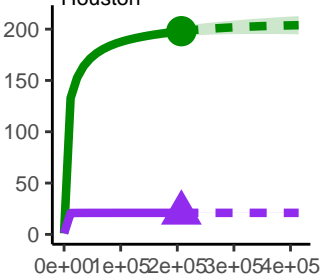

Supplement: Figure 2—source data 1. — This zipped file includes plots for the tree community of each city, showing rarefaction curves as calculated by the R package iNext. Each city includes a plot for all trees and a plot for all naturally occurring trees. [file elife-77891-fig2-data1.zip › Rarefaction_Plots/Houston_RarefactionPlot_Diversity_all.pdf]

# Houston

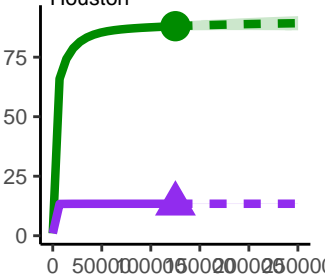

Supplement: Figure 2—source data 1. — This zipped file includes plots for the tree community of each city, showing rarefaction curves as calculated by the R package iNext. Each city includes a plot for all trees and a plot for all naturally occurring trees. [file elife-77891-fig2-data1.zip › Rarefaction_Plots/Houston_RarefactionPlot_Diversity_native.pdf]

# HuntingtonBeach

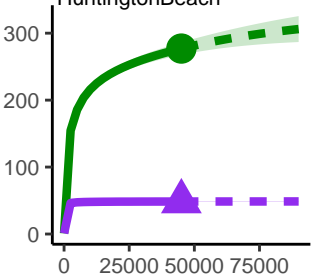

Supplement: Figure 2—source data 1. — This zipped file includes plots for the tree community of each city, showing rarefaction curves as calculated by the R package iNext. Each city includes a plot for all trees and a plot for all naturally occurring trees. [file elife-77891-fig2-data1.zip › Rarefaction_Plots/Huntington Beach_RarefactionPlot_Diversity_all.pdf]

# HuntingtonBeach

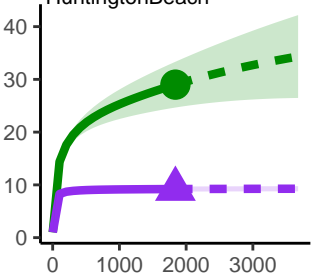

Supplement: Figure 2—source data 1. — This zipped file includes plots for the tree community of each city, showing rarefaction curves as calculated by the R package iNext. Each city includes a plot for all trees and a plot for all naturally occurring trees. [file elife-77891-fig2-data1.zip › Rarefaction_Plots/Huntington Beach_RarefactionPlot_Diversity_native.pdf]

# Indianapolis

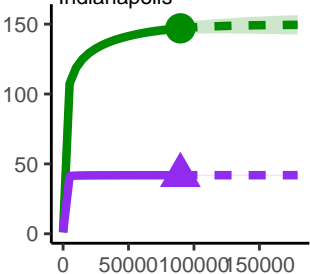

Supplement: Figure 2—source data 1. — This zipped file includes plots for the tree community of each city, showing rarefaction curves as calculated by the R package iNext. Each city includes a plot for all trees and a plot for all naturally occurring trees. [file elife-77891-fig2-data1.zip › Rarefaction_Plots/Indianapolis_RarefactionPlot_Diversity_all.pdf]

# Indianapolis

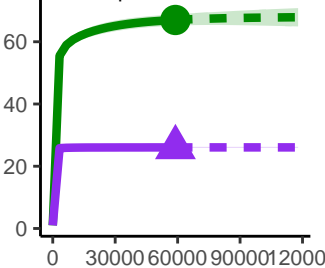

Supplement: Figure 2—source data 1. — This zipped file includes plots for the tree community of each city, showing rarefaction curves as calculated by the R package iNext. Each city includes a plot for all trees and a plot for all naturally occurring trees. [file elife-77891-fig2-data1.zip › Rarefaction_Plots/Indianapolis_RarefactionPlot_Diversity_native.pdf]

Irvine

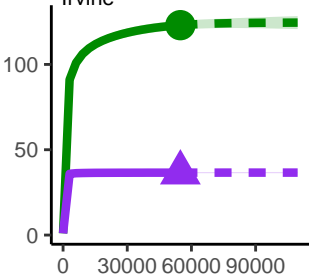

Supplement: Figure 2—source data 1. — This zipped file includes plots for the tree community of each city, showing rarefaction curves as calculated by the R package iNext. Each city includes a plot for all trees and a plot for all naturally occurring trees. [file elife-77891-fig2-data1.zip › Rarefaction_Plots/Irvine_RarefactionPlot_Diversity_all.pdf]

Irvine

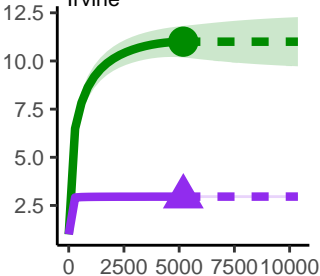

Supplement: Figure 2—source data 1. — This zipped file includes plots for the tree community of each city, showing rarefaction curves as calculated by the R package iNext. Each city includes a plot for all trees and a plot for all naturally occurring trees. [file elife-77891-fig2-data1.zip › Rarefaction_Plots/Irvine_RarefactionPlot_Diversity_native.pdf]

# Jerseycity

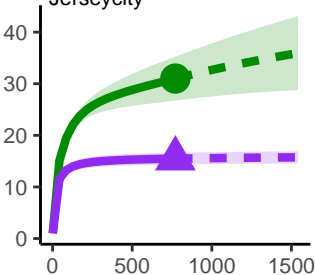

Supplement: Figure 2—source data 1. — This zipped file includes plots for the tree community of each city, showing rarefaction curves as calculated by the R package iNext. Each city includes a plot for all trees and a plot for all naturally occurring trees. [file elife-77891-fig2-data1.zip › Rarefaction_Plots/Jersey City_RarefactionPlot_Diversity_all.pdf]

Jerseycity

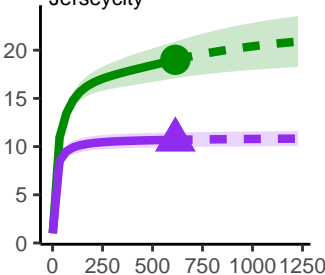

Supplement: Figure 2—source data 1. — This zipped file includes plots for the tree community of each city, showing rarefaction curves as calculated by the R package iNext. Each city includes a plot for all trees and a plot for all naturally occurring trees. [file elife-77891-fig2-data1.zip › Rarefaction_Plots/Jersey City_RarefactionPlot_Diversity_native.pdf]

## Knoxville

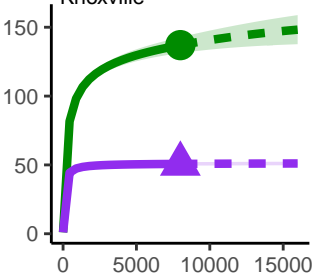

Supplement: Figure 2—source data 1. — This zipped file includes plots for the tree community of each city, showing rarefaction curves as calculated by the R package iNext. Each city includes a plot for all trees and a plot for all naturally occurring trees. [file elife-77891-fig2-data1.zip › Rarefaction_Plots/Knoxville_RarefactionPlot_Diversity_all.pdf]

## Knoxville

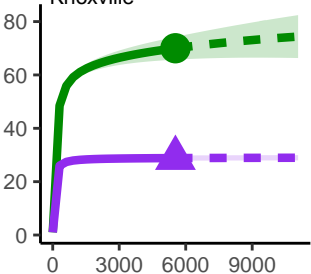

Supplement: Figure 2—source data 1. — This zipped file includes plots for the tree community of each city, showing rarefaction curves as calculated by the R package iNext. Each city includes a plot for all trees and a plot for all naturally occurring trees. [file elife-77891-fig2-data1.zip › Rarefaction_Plots/Knoxville_RarefactionPlot_Diversity_native.pdf]

Las Vegas

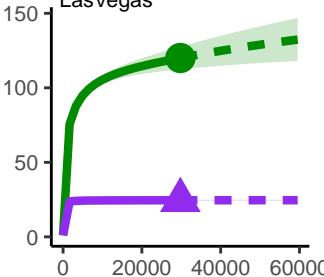

Supplement: Figure 2—source data 1. — This zipped file includes plots for the tree community of each city, showing rarefaction curves as calculated by the R package iNext. Each city includes a plot for all trees and a plot for all naturally occurring trees. [file elife-77891-fig2-data1.zip › Rarefaction_Plots/Las Vegas_RarefactionPlot_Diversity_all.pdf]

Las Vegas

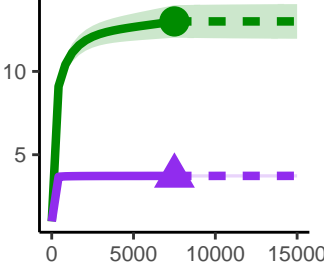

Supplement: Figure 2—source data 1. — This zipped file includes plots for the tree community of each city, showing rarefaction curves as calculated by the R package iNext. Each city includes a plot for all trees and a plot for all naturally occurring trees. [file elife-77891-fig2-data1.zip › Rarefaction_Plots/Las Vegas_RarefactionPlot_Diversity_native.pdf]

LosAngeles

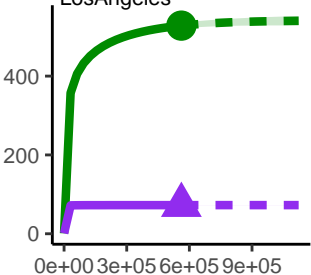

Supplement: Figure 2—source data 1. — This zipped file includes plots for the tree community of each city, showing rarefaction curves as calculated by the R package iNext. Each city includes a plot for all trees and a plot for all naturally occurring trees. [file elife-77891-fig2-data1.zip › Rarefaction_Plots/Los Angeles_RarefactionPlot_Diversity_all.pdf]

LosAngeles

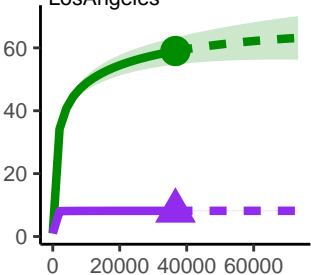

Supplement: Figure 2—source data 1. — This zipped file includes plots for the tree community of each city, showing rarefaction curves as calculated by the R package iNext. Each city includes a plot for all trees and a plot for all naturally occurring trees. [file elife-77891-fig2-data1.zip › Rarefaction_Plots/Los Angeles_RarefactionPlot_Diversity_native.pdf]

# Louisville

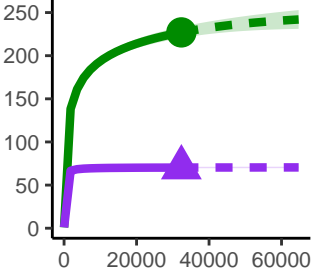

Supplement: Figure 2—source data 1. — This zipped file includes plots for the tree community of each city, showing rarefaction curves as calculated by the R package iNext. Each city includes a plot for all trees and a plot for all naturally occurring trees. [file elife-77891-fig2-data1.zip › Rarefaction_Plots/Louisville_RarefactionPlot_Diversity_all.pdf]

Louisville

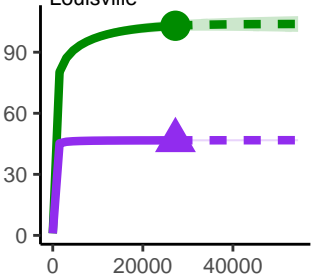

Supplement: Figure 2—source data 1. — This zipped file includes plots for the tree community of each city, showing rarefaction curves as calculated by the R package iNext. Each city includes a plot for all trees and a plot for all naturally occurring trees. [file elife-77891-fig2-data1.zip › Rarefaction_Plots/Louisville_RarefactionPlot_Diversity_native.pdf]

Madison

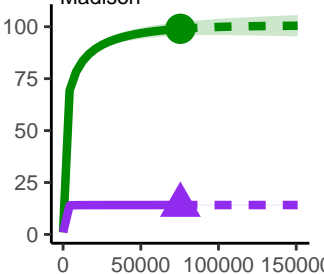

Supplement: Figure 2—source data 1. — This zipped file includes plots for the tree community of each city, showing rarefaction curves as calculated by the R package iNext. Each city includes a plot for all trees and a plot for all naturally occurring trees. [file elife-77891-fig2-data1.zip › Rarefaction_Plots/Madison_RarefactionPlot_Diversity_all.pdf]

Madison

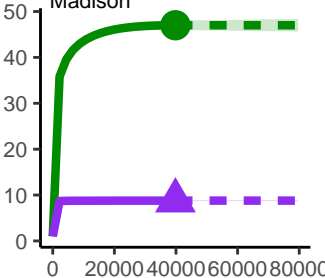

Supplement: Figure 2—source data 1. — This zipped file includes plots for the tree community of each city, showing rarefaction curves as calculated by the R package iNext. Each city includes a plot for all trees and a plot for all naturally occurring trees. [file elife-77891-fig2-data1.zip › Rarefaction_Plots/Madison_RarefactionPlot_Diversity_native.pdf]

Miami

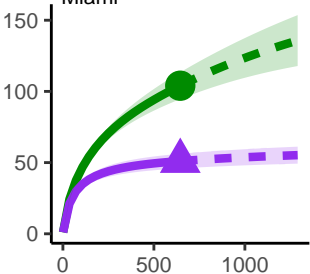

Supplement: Figure 2—source data 1. — This zipped file includes plots for the tree community of each city, showing rarefaction curves as calculated by the R package iNext. Each city includes a plot for all trees and a plot for all naturally occurring trees. [file elife-77891-fig2-data1.zip › Rarefaction_Plots/Miami_RarefactionPlot_Diversity_all.pdf]

Miami

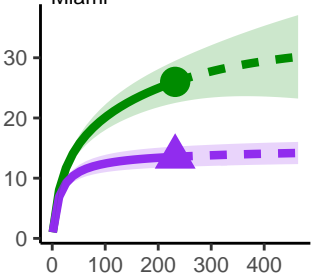

Supplement: Figure 2—source data 1. — This zipped file includes plots for the tree community of each city, showing rarefaction curves as calculated by the R package iNext. Each city includes a plot for all trees and a plot for all naturally occurring trees. [file elife-77891-fig2-data1.zip › Rarefaction_Plots/Miami_RarefactionPlot_Diversity_native.pdf]

# Milwaukee

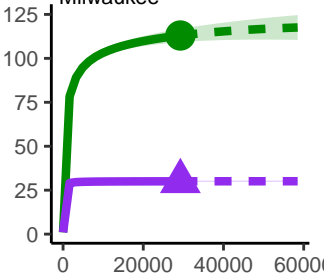

Supplement: Figure 2—source data 1. — This zipped file includes plots for the tree community of each city, showing rarefaction curves as calculated by the R package iNext. Each city includes a plot for all trees and a plot for all naturally occurring trees. [file elife-77891-fig2-data1.zip › Rarefaction_Plots/Milwaukee_RarefactionPlot_Diversity_all.pdf]

Milwaukee

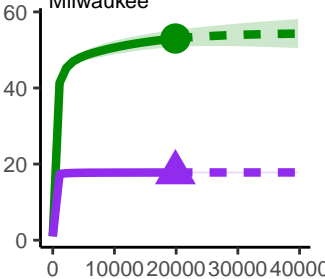

Supplement: Figure 2—source data 1. — This zipped file includes plots for the tree community of each city, showing rarefaction curves as calculated by the R package iNext. Each city includes a plot for all trees and a plot for all naturally occurring trees. [file elife-77891-fig2-data1.zip › Rarefaction_Plots/Milwaukee_RarefactionPlot_Diversity_native.pdf]

Minneapolis

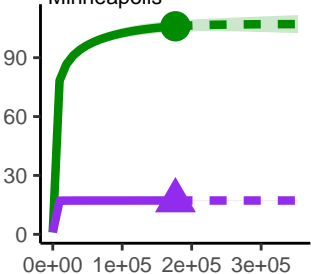

Supplement: Figure 2—source data 1. — This zipped file includes plots for the tree community of each city, showing rarefaction curves as calculated by the R package iNext. Each city includes a plot for all trees and a plot for all naturally occurring trees. [file elife-77891-fig2-data1.zip › Rarefaction_Plots/Minneapolis_RarefactionPlot_Diversity_all.pdf]

# Minneapolis

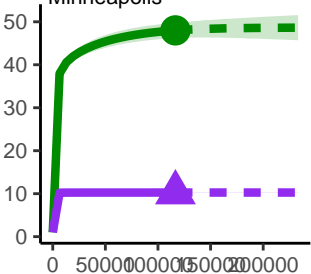

Supplement: Figure 2—source data 1. — This zipped file includes plots for the tree community of each city, showing rarefaction curves as calculated by the R package iNext. Each city includes a plot for all trees and a plot for all naturally occurring trees. [file elife-77891-fig2-data1.zip › Rarefaction_Plots/Minneapolis_RarefactionPlot_Diversity_native.pdf]

# Nashville

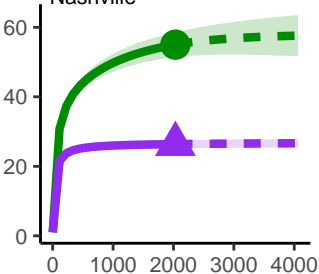

Supplement: Figure 2—source data 1. — This zipped file includes plots for the tree community of each city, showing rarefaction curves as calculated by the R package iNext. Each city includes a plot for all trees and a plot for all naturally occurring trees. [file elife-77891-fig2-data1.zip › Rarefaction_Plots/Nashville_RarefactionPlot_Diversity_all.pdf]

# Nashville

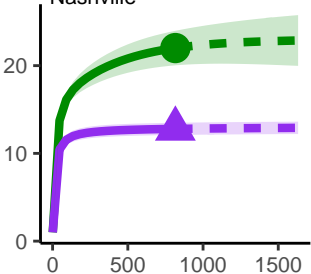

Supplement: Figure 2—source data 1. — This zipped file includes plots for the tree community of each city, showing rarefaction curves as calculated by the R package iNext. Each city includes a plot for all trees and a plot for all naturally occurring trees. [file elife-77891-fig2-data1.zip › Rarefaction_Plots/Nashville_RarefactionPlot_Diversity_native.pdf]

## New Orleans

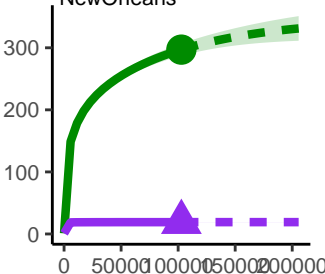

Supplement: Figure 2—source data 1. — This zipped file includes plots for the tree community of each city, showing rarefaction curves as calculated by the R package iNext. Each city includes a plot for all trees and a plot for all naturally occurring trees. [file elife-77891-fig2-data1.zip › Rarefaction_Plots/New Orleans_RarefactionPlot_Diversity_all.pdf]

# NewOrleans

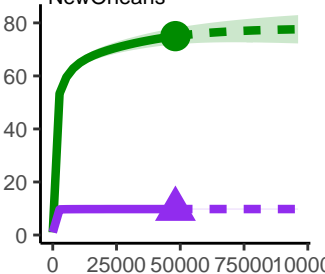

Supplement: Figure 2—source data 1. — This zipped file includes plots for the tree community of each city, showing rarefaction curves as calculated by the R package iNext. Each city includes a plot for all trees and a plot for all naturally occurring trees. [file elife-77891-fig2-data1.zip › Rarefaction_Plots/New Orleans_RarefactionPlot_Diversity_native.pdf]

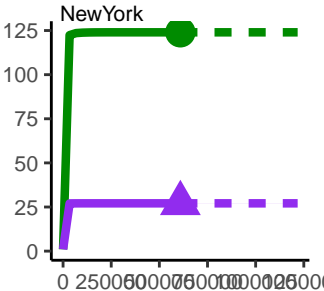

Supplement: Figure 2—source data 1. — This zipped file includes plots for the tree community of each city, showing rarefaction curves as calculated by the R package iNext. Each city includes a plot for all trees and a plot for all naturally occurring trees. [file elife-77891-fig2-data1.zip › Rarefaction_Plots/New York_RarefactionPlot_Diversity_all.pdf]

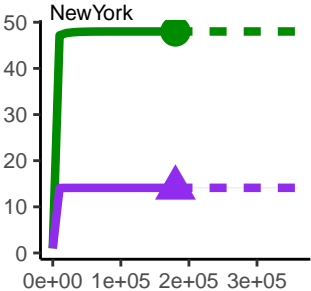

Supplement: Figure 2—source data 1. — This zipped file includes plots for the tree community of each city, showing rarefaction curves as calculated by the R package iNext. Each city includes a plot for all trees and a plot for all naturally occurring trees. [file elife-77891-fig2-data1.zip › Rarefaction_Plots/New York_RarefactionPlot_Diversity_native.pdf]

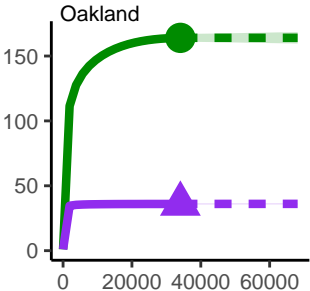

Supplement: Figure 2—source data 1. — This zipped file includes plots for the tree community of each city, showing rarefaction curves as calculated by the R package iNext. Each city includes a plot for all trees and a plot for all naturally occurring trees. [file elife-77891-fig2-data1.zip › Rarefaction_Plots/Oakland_RarefactionPlot_Diversity_all.pdf]

Oakland

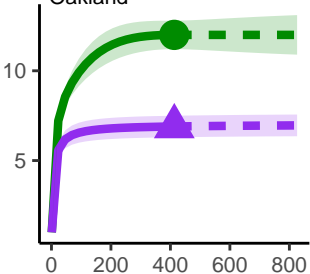

Supplement: Figure 2—source data 1. — This zipped file includes plots for the tree community of each city, showing rarefaction curves as calculated by the R package iNext. Each city includes a plot for all trees and a plot for all naturally occurring trees. [file elife-77891-fig2-data1.zip › Rarefaction_Plots/Oakland_RarefactionPlot_Diversity_native.pdf]

# OklahomaCity

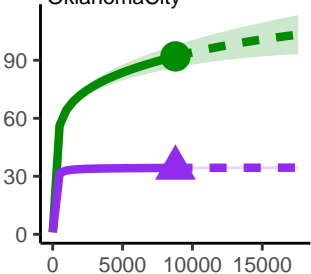

Supplement: Figure 2—source data 1. — This zipped file includes plots for the tree community of each city, showing rarefaction curves as calculated by the R package iNext. Each city includes a plot for all trees and a plot for all naturally occurring trees. [file elife-77891-fig2-data1.zip › Rarefaction_Plots/Oklahoma City_RarefactionPlot_Diversity_all.pdf]

# OklahomaCity

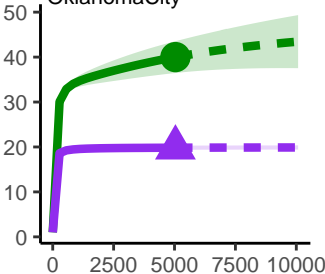

Supplement: Figure 2—source data 1. — This zipped file includes plots for the tree community of each city, showing rarefaction curves as calculated by the R package iNext. Each city includes a plot for all trees and a plot for all naturally occurring trees. [file elife-77891-fig2-data1.zip › Rarefaction_Plots/Oklahoma City_RarefactionPlot_Diversity_native.pdf]

# Ontario

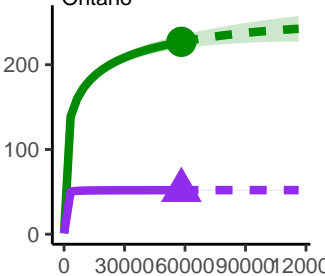

Supplement: Figure 2—source data 1. — This zipped file includes plots for the tree community of each city, showing rarefaction curves as calculated by the R package iNext. Each city includes a plot for all trees and a plot for all naturally occurring trees. [file elife-77891-fig2-data1.zip › Rarefaction_Plots/Ontario_RarefactionPlot_Diversity_all.pdf]

# Ontario

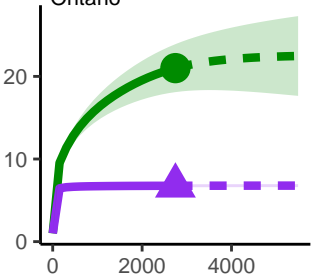

Supplement: Figure 2—source data 1. — This zipped file includes plots for the tree community of each city, showing rarefaction curves as calculated by the R package iNext. Each city includes a plot for all trees and a plot for all naturally occurring trees. [file elife-77891-fig2-data1.zip › Rarefaction_Plots/Ontario_RarefactionPlot_Diversity_native.pdf]

Orlando

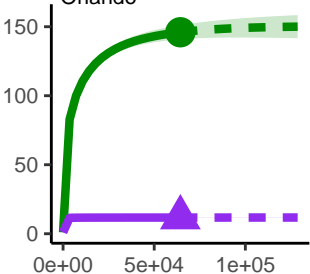

Supplement: Figure 2—source data 1. — This zipped file includes plots for the tree community of each city, showing rarefaction curves as calculated by the R package iNext. Each city includes a plot for all trees and a plot for all naturally occurring trees. [file elife-77891-fig2-data1.zip › Rarefaction_Plots/Orlando_RarefactionPlot_Diversity_all.pdf]

## Orlando

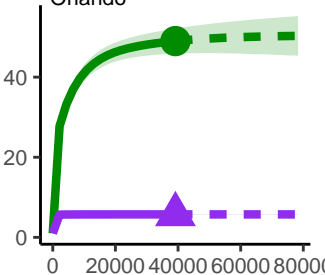

Supplement: Figure 2—source data 1. — This zipped file includes plots for the tree community of each city, showing rarefaction curves as calculated by the R package iNext. Each city includes a plot for all trees and a plot for all naturally occurring trees. [file elife-77891-fig2-data1.zip › Rarefaction_Plots/Orlando_RarefactionPlot_Diversity_native.pdf]

# OverlandPark

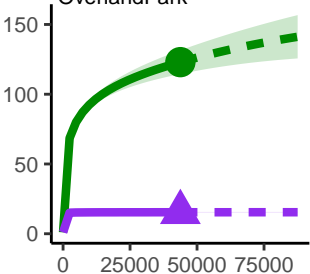

Supplement: Figure 2—source data 1. — This zipped file includes plots for the tree community of each city, showing rarefaction curves as calculated by the R package iNext. Each city includes a plot for all trees and a plot for all naturally occurring trees. [file elife-77891-fig2-data1.zip › Rarefaction_Plots/Overland Park_RarefactionPlot_Diversity_all.pdf]

# OverlandPark

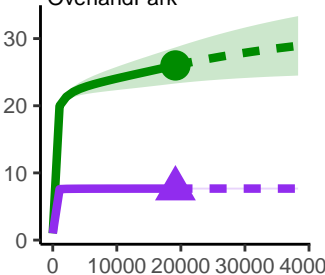

Supplement: Figure 2—source data 1. — This zipped file includes plots for the tree community of each city, showing rarefaction curves as calculated by the R package iNext. Each city includes a plot for all trees and a plot for all naturally occurring trees. [file elife-77891-fig2-data1.zip › Rarefaction_Plots/Overland Park_RarefactionPlot_Diversity_native.pdf]

# Phoenix

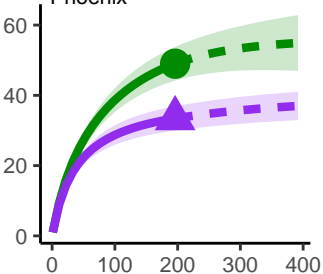

Supplement: Figure 2—source data 1. — This zipped file includes plots for the tree community of each city, showing rarefaction curves as calculated by the R package iNext. Each city includes a plot for all trees and a plot for all naturally occurring trees. [file elife-77891-fig2-data1.zip › Rarefaction_Plots/Phoenix_RarefactionPlot_Diversity_all.pdf]

Phoenix

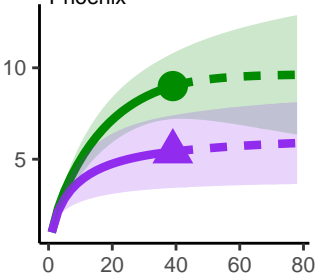

Supplement: Figure 2—source data 1. — This zipped file includes plots for the tree community of each city, showing rarefaction curves as calculated by the R package iNext. Each city includes a plot for all trees and a plot for all naturally occurring trees. [file elife-77891-fig2-data1.zip › Rarefaction_Plots/Phoenix_RarefactionPlot_Diversity_native.pdf]

# Pittsburgh

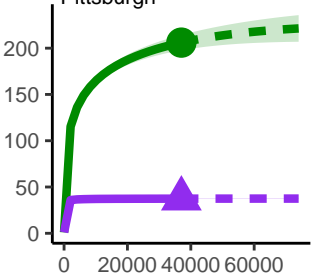

Supplement: Figure 2—source data 1. — This zipped file includes plots for the tree community of each city, showing rarefaction curves as calculated by the R package iNext. Each city includes a plot for all trees and a plot for all naturally occurring trees. [file elife-77891-fig2-data1.zip › Rarefaction_Plots/Pittsburgh_RarefactionPlot_Diversity_all.pdf]

# Pittsburgh

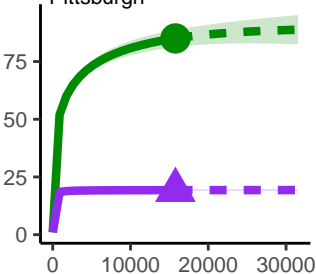

Supplement: Figure 2—source data 1. — This zipped file includes plots for the tree community of each city, showing rarefaction curves as calculated by the R package iNext. Each city includes a plot for all trees and a plot for all naturally occurring trees. [file elife-77891-fig2-data1.zip › Rarefaction_Plots/Pittsburgh_RarefactionPlot_Diversity_native.pdf]

Plano

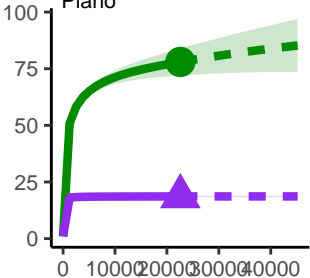

Supplement: Figure 2—source data 1. — This zipped file includes plots for the tree community of each city, showing rarefaction curves as calculated by the R package iNext. Each city includes a plot for all trees and a plot for all naturally occurring trees. [file elife-77891-fig2-data1.zip › Rarefaction_Plots/Plano_RarefactionPlot_Diversity_all.pdf]

Plano

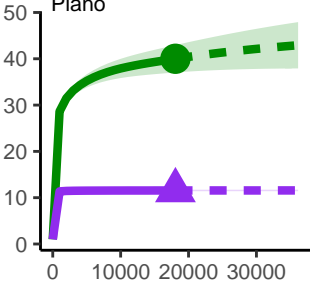

Supplement: Figure 2—source data 1. — This zipped file includes plots for the tree community of each city, showing rarefaction curves as calculated by the R package iNext. Each city includes a plot for all trees and a plot for all naturally occurring trees. [file elife-77891-fig2-data1.zip › Rarefaction_Plots/Plano_RarefactionPlot_Diversity_native.pdf]

Portland

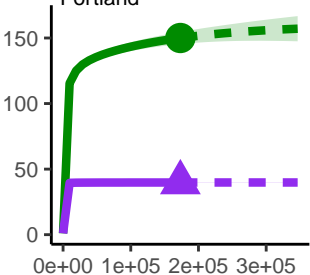

Supplement: Figure 2—source data 1. — This zipped file includes plots for the tree community of each city, showing rarefaction curves as calculated by the R package iNext. Each city includes a plot for all trees and a plot for all naturally occurring trees. [file elife-77891-fig2-data1.zip › Rarefaction_Plots/Portland_RarefactionPlot_Diversity_all.pdf]

## Portland

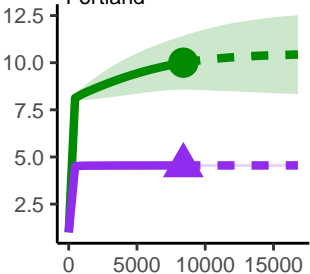

Supplement: Figure 2—source data 1. — This zipped file includes plots for the tree community of each city, showing rarefaction curves as calculated by the R package iNext. Each city includes a plot for all trees and a plot for all naturally occurring trees. [file elife-77891-fig2-data1.zip › Rarefaction_Plots/Portland_RarefactionPlot_Diversity_native.pdf]

# Providence

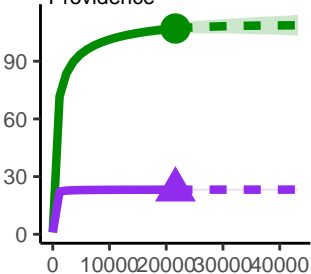

Supplement: Figure 2—source data 1. — This zipped file includes plots for the tree community of each city, showing rarefaction curves as calculated by the R package iNext. Each city includes a plot for all trees and a plot for all naturally occurring trees. [file elife-77891-fig2-data1.zip › Rarefaction_Plots/Providence_RarefactionPlot_Diversity_all.pdf]

# RanchoCucamonga

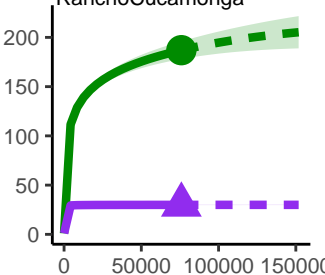

Supplement: Figure 2—source data 1. — This zipped file includes plots for the tree community of each city, showing rarefaction curves as calculated by the R package iNext. Each city includes a plot for all trees and a plot for all naturally occurring trees. [file elife-77891-fig2-data1.zip › Rarefaction_Plots/Rancho Cucamonga_RarefactionPlot_Diversity_all.pdf]

# RanchoCucamonga

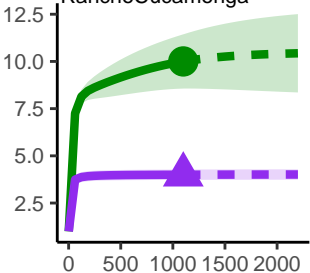

Supplement: Figure 2—source data 1. — This zipped file includes plots for the tree community of each city, showing rarefaction curves as calculated by the R package iNext. Each city includes a plot for all trees and a plot for all naturally occurring trees. [file elife-77891-fig2-data1.zip › Rarefaction_Plots/Rancho Cucamonga_RarefactionPlot_Diversity_native.pdf]

# Richmond

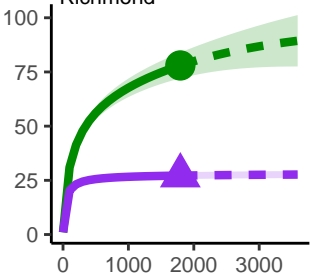

Supplement: Figure 2—source data 1. — This zipped file includes plots for the tree community of each city, showing rarefaction curves as calculated by the R package iNext. Each city includes a plot for all trees and a plot for all naturally occurring trees. [file elife-77891-fig2-data1.zip › Rarefaction_Plots/Richmond_RarefactionPlot_Diversity_all.pdf]

# Richmond

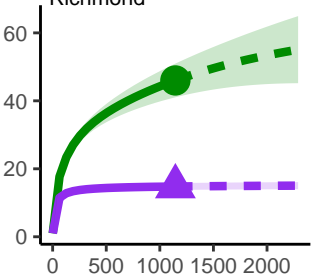

Supplement: Figure 2—source data 1. — This zipped file includes plots for the tree community of each city, showing rarefaction curves as calculated by the R package iNext. Each city includes a plot for all trees and a plot for all naturally occurring trees. [file elife-77891-fig2-data1.zip › Rarefaction_Plots/Richmond_RarefactionPlot_Diversity_native.pdf]

Rochester

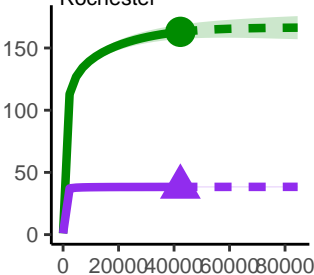

Supplement: Figure 2—source data 1. — This zipped file includes plots for the tree community of each city, showing rarefaction curves as calculated by the R package iNext. Each city includes a plot for all trees and a plot for all naturally occurring trees. [file elife-77891-fig2-data1.zip › Rarefaction_Plots/Rochester_RarefactionPlot_Diversity_all.pdf]
