# Supplementary material for: Species clustering, climate effects, and introduced species in 5 million city trees across 63 US cities
Source: eLife. 2022 Sep 27;11:e77891. doi: 10.7554/eLife.77891 (PMC9578703; doi:10.7554/eLife.77891)
Supplement: Figure 2—source data 1. — This zipped file includes plots for the tree community of each city, showing rarefaction curves as calculated by the R package iNext. Each city includes a plot for all trees and a plot for all naturally occurring trees. [file Providence_RarefactionPlot_Diversity_native.pdf › Rarefaction_Plots/Providence_RarefactionPlot_Diversity_native.pdf]

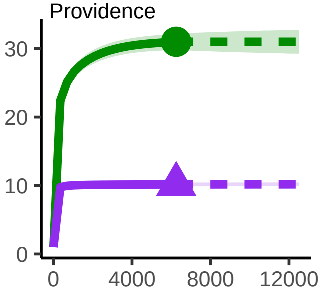

Providence
30
20
10
0
0
4000
8000
12000
